# Is rectal MRI beneficial for determining the location of rectal cancer with respect to the peritoneal reflection?

**DOI:** 10.2478/v10019-012-0038-7

**Published:** 2012-11-09

**Authors:** Eun Joo Jung, Chun Geun Ryu, Gangmi Kim, Su Ran Kim, Sang Eun Nam, Hee Sun Park, Young Jun Kim, Dae-Yong Hwang

**Affiliations:** 1 Department of Surgery, Colorectal Cancer Center, Konkuk University Medical Center, Konkuk University School of Medicine, Seoul, Republic of Korea; 2 Department of Radiology, Konkuk University Medical Center, Konkuk University School of Medicine, Seoul, Republic of Korea

**Keywords:** rectal cancer, peritoneal reflection, magnetic resonance imaging (MRI)

## Abstract

**Background:**

An objective method for determining the location of the cancer with respect to peritoneal reflection would be helpful to decide the treatment modality for rectal cancer. This study was designed to evaluate the accuracy and usefulness of rectal MRI to determine spatial relations between the peritoneal reflection and rectal cancer and to compare these with operative findings.

**Patients and methods:**

Patients that underwent a rectal cancer operation after a rectal MRI check between November 2008 and June 2010 were considered for the study. The patients that received preoperative concurrent chemoradiation or trans-anal local excision were excluded.

**Results:**

Fifty-four patients constituted the study cohort. By comparing surgical and radiologic findings, the accuracy for predicting tumour location in relation to the peritoneal reflection by rectal MRI in all patients was 90.7%. In terms of tumour location in relation to peritoneal reflection, the accuracy of rectal MRI was 93.5% in patients with a tumour located above the peritoneal reflection, 90.0% in patients with a tumour located on the peritoneal reflection, and 84.6% in patients with a tumour located below the peritoneal reflection (p=0.061). When the cohort was subdivided by gender, body mass index (BMI), operative findings, or tumour size, no significant difference was observed among subgroups.

**Conclusions:**

Rectal MRI could be a useful tool for evaluating the relation between rectal cancer and peritoneal reflection especially when tumour size is less than 8cm. Rectal MRI can provide information regarding the location of rectal cancer in relation to the peritoneal reflection for treatment planning purposes.

## Introduction

Preoperative evaluations are of considerable importance for rectal cancer management, because treatment decision making is dependent on radiologic findings. Thus, neoadjuvant therapy could be determined based on preoperative clinical staging status, but it has not been determined which parts of rectal tumours should be included in such as staging system. Some authors have suggested that considerations of height from the anal verge might have beneficial on the radiotherapy of rectal tumors.^1^ However, measurements of distances from the anal verge are unclear because the methods devised to date, *e.g*., digital rectal examination or even rigid sigmoidoscopy, are rather subjective.

The peritoneal reflection is a landmark used for evaluating the rectum anteriorly, and divides the rectum into two parts, that is, the intraperitoneal and extraperitoneal regions, which are referred to considerations of the venous and lymphatic drainage systems of the rectum.^2^ In particular, extraperitoneal rectal tumours disseminate mainly through the systemic pelvic venous and lateral lymphatic drainage systems, whereas intraperitoneal rectal tumours disseminate mainly through the superior haemorrhoidal and inferior mesenteric venous and lymphatic drainage systems.^2^ Furthermore, Benzoni *et al.* concluded that tumour location in relation to the peritoneal reflection is a prognostic factor in rectal cancer.^2^ In this study, it was found that extraperitoneal rectal tumours are more aggressive than intraperitoneal tumours, even when treated by neoadjuvant chemoradiotherapy before surgery, which is common approach in the treatment of rectal cancer.^2^–^4^ Thus, it appears that the peritoneal reflection might be useful for the adaptation of different treatment strategies in rectal cancer. Even though the definitions of intraperitoneal and extraperitoneal locations are ambiguous in relation to the mesorectum, it may be that the use of the peritoneal reflection as a discriminating structure in the pelvic cavity enables the differentiation of the locations of rectal tumours to the intraperitoneal and extraperitoneal regions.

Reported distances from the anal verge to the peritoneal reflection are highly variable.^1^,^5^ Accordingly, measurements based on this landmark cannot be used to precisely determine the location of the peritoneal reflection. We considered that if the peritoneal reflection could be clearly visualized and localized radiologically, that a more objective localization method could be devised than those based on distances from the anal verge.

Many reports have been issued on the role of magnetic resonance imaging (MRI) in rectal cancer in terms of determining circumferential margins or perirectal nodal statuses.^6^–^8^ However, few reports are available on the spatial relation between rectal cancer and the peritoneal reflection as determined by rectal MRI. Therefore, the aim of this study was to evaluate the accuracy and usefulness of rectal MRI for determining the relation between rectal cancer and the peritoneal reflection with respect to operative findings.

## Materials and methods

Of the patients that underwent surgery for rectal cancer after a preoperative work-up (including rectal MRI) at the Colorectal Cancer Center, Konkuk University Medical Center between November 2008 and June 2010, 54 patients that did not receive preoperative concurrent chemoradiation or trans-anal local excision were included in the present study.

**FIGURE 1.**
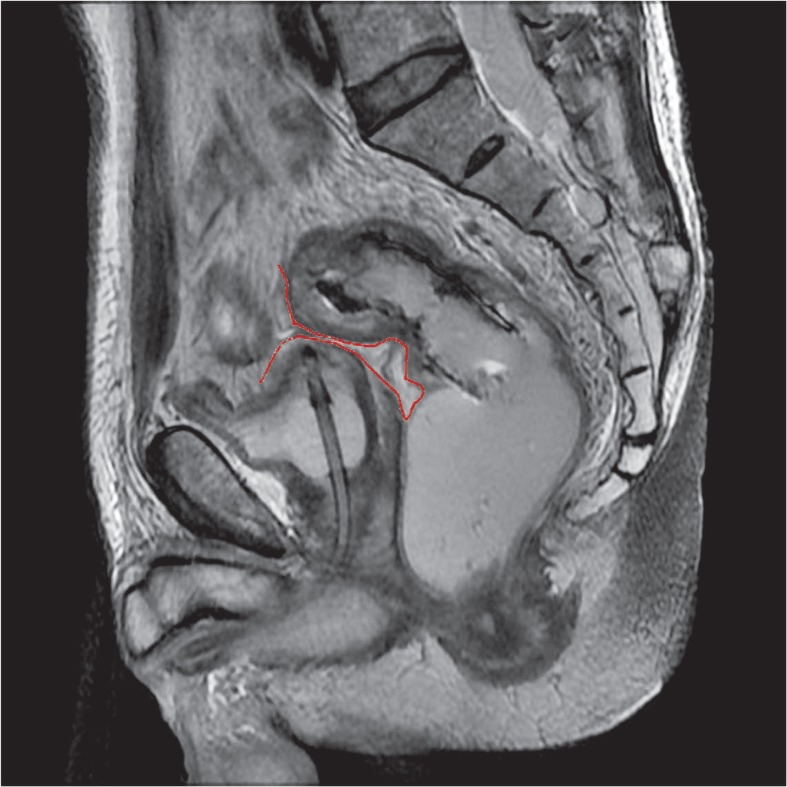
Sagittal view of the peritoneal reflection (red line) by rectal MRI.

**FIGURE 2.**
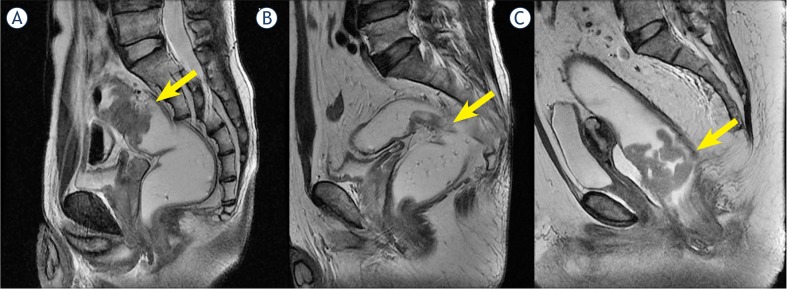
Tumour location with respect to the peritoneal reflection (PR). a. Tumour above the PR; b. tumour at the PR; c. tumour below the PR.

Rectal MRI images were reviewed by radiologists (H.S.P., and Y.J.K), on axial, sagittal, and coronal scans of T2-weighted images without clinical information. The peritoneal reflection appears as a low-signal-intensity linear structure that extends over the surface of the bladder and can be traced posteriorly to its point of attachment onto the rectum^6^ (Figure 1). Under the consensus decision, these two radiologists determined spatial relationships between rectal cancer and the peritoneal reflection, and allocated tumour locations to the following categories; a) a location completely proximal to the peritoneal reflection, b) a location mainly at the level of the peritoneal reflection, c) a location completely distal to the peritoneal reflection (Figure 2). In addition, tumour locations were categorized as; anterior, lateral, posterior, circumferential, anterolateral, or postero lateral.

Operative findings were recorded by a colorectal surgeon (DYH), who performed all surgeries. Intraoperative tumour levels were described by the surgeon as above, on, or below the peritoneal reflection, and as anterior, posterior, lateral, or circumferential. Tumour sizes were determined pathologically and tumours were staged according to the TNM staging system. Distances from the anal verge to lower tumour borders were determined by digital rectal examination and by sigmoidoscopy.

Body mass index (BMI) was calculated using weight (kg) divided by height (m) squared, and was divided into three groups; the low BMI group (<20 kg/m^2^), the normal BMI group (≥20 and <25 kg/m^2^), and the high BMI group (≥25 kg/m^2^).

Data analysis was performed using the ‘Statistical Package for the Social Sciences (SPSS)’ version 14.0 for Windows (SPSS, Inc. Chicago, IL). Pearson’s chi-square test was used to compare locational accuracies between subgroups, and *p*-values of <0.05 were considered statistically significant.

## Results

The 54 study subjects (32 males and 22 females) had a mean age of 62.2 years. All were diagnosed with adenocarcinoma and low anterior resection was performed in 49 (90.7%) and abdominoperineal resection in 5 (9.3%). TNM tumour stage was 0 in 2 patients (3.7%), I in 11 patients (20.4%), II in 17 patients (31.5%), III in 22 patients (40.7%), and IV in 2 patients (3.7%). Mean tumour size was 4.8 cm. Patients’ characteristics are summarized in Table 1.

The accuracy of predicting tumour location relative to the peritoneal reflection by rectal MRI using surgical findings as the standard in all 54 patients was 90.7% (Table 2). No significant differences were found between gender, BMI, operative findings, and tumour size subgroups. The accuracy of predicting tumour location relative to the peritoneal reflection was 90.9% for males and 87.5% for females (*p*=0.092); for BMI, accuracies were 88.0%, 91.6 %, and 86.3% in the low, normal, and high BMI subgroups, respectively (*p*=0.528).

The accuracy of rectal MRI was 93.5% in patients with a tumour located above the peritoneal reflection, 90.0% in patients with a tumour located on the peritoneal reflection, and 84.6% in patients with tumour located below the peritoneal reflection (*p*=0.061). When tumours were classified by 2 cm increments in size, accuracies were 88.9% for a tumour size of 0–1.9 cm, 91.7% for 2.0–3.9cm, 93.3% for 4.0–5.9 cm, 100% for 6.0–7.9 cm, and 57.1% for 8.0–10.0 cm (*p*=0.394), indicating that accuracy increased with tumour size until tumours exceeded 8 cm.

In terms of predicting tumour direction (anterior, lateral, and posterior), the overall accuracy of rectal MRI was 44.4%. No significant difference was observed between gender, BMI, operative findings, and tumour size subgroups. Overall, tumour directions were predicted less accurately than tumour locations (Table 3).

## Discussions

When considering treatment options for rectal cancer, preoperative evaluations are important, because decisions regarding surgery and preoperative concurrent chemoradiotherapy are dependent on tumour location, mesorectal fascia involvement, and nodal status.^6^,^7^,^9^–^13^ Rectal MRI is commonly performed preoperatively for evaluating the mesorectal fascia or adjacent organ involvement, and nodal staging.^6^–^9^ However, few reports have described the clinical usefulness of rectal MRI in terms of evaluating spatial relations between rectal tumours and the peritoneal reflection.

For descriptive purposes, the rectum is divided into three parts, that is, the upper, mid, and lower thirds. The upper third is covered by peritoneum anteriorly and laterally, whereas the middle third is covered only anteriorly, and the lower third is devoid of peritoneum.^9^,^10^ In Japanese classification, the rectum is also divided into three parts, designated Rs, Ra, and Rb.^14^ The border between Ra and Rb is defined to be at the level of the peritoneal reflection, which approximately corresponds to the level of the middle Houston valve.^14^ Thus, these classifications are based on the relation with respect to peritoneal reflection. However, it is not easy to determine the precise location of the peritoneal reflection preoperatively.

For this reason, most articles on rectal cancer define the upper rectum as 10 to 15cm from the anal verge, the mid third as 5 to 10 cm, and the lower third as < 5cm, although one author defined the upper third as 12 to 16cm from the anal verge.^15^ However, this classification is vague and subjective, and reported distances from the anal verge based on these arbitrary divisions are not comparable.

Accordingly, we considered that the peritoneal reflection might be of use as a landmark to determine the location of the rectal subdivision for rectal cancer management. Some authors have evaluated the use of the peritoneal reflection as an anatomic landmark in rectal cancer patients. Gerdes *et al.* used trans-endorectal ultrasound (TRUS) to evaluate tumour locations with respect to the peritoneal reflection.^16^ In this study, the indicators of an intraperitoneal location were peristalsis beyond the rectal wall or intraperitoneal fluid collection.^16^ However, the study had two limitations, namely, that the peritoneal reflection could not be found in the absence of bowel peristalsis or fluid collection, and that trans-endorectal ultrasound is a practitioner-dependent subjective procedure. Others have also evaluated the usefulness of the peritoneal reflection as a landmark in rectal cancer by comparing intraoperative rigid proctoscopy and intraoperative findings.^5^,^17^ However, in these studies, data just suggested the location of peritoneal reflection or distance between the peritoneal reflection and anal verge, and in clinical practice, this data is not applicable for determining treatment plans in rectal cancer.

On rectal MR images, the peritoneal reflection appears as low-signal intensity linear structure at the junction between the rectum and the posterior aspect of the bladder in males or the vagina in females (Figure 1). In the present study, the accuracy of predicting the location of a rectal tumour with respect to the peritoneal reflection exceeded 88%. Furthermore, clinical variables examined, such as gender or BMI, had no effect on this accuracy, but when the tumour size exceeded 8 cm, accuracy fell to 57%, presumably because large tumours disrupt the normal anatomy.

In the present study, the accuracy of determining tumour direction was not high as 44.4%, probably because natural rectal folds make the interpretation of direction difficult, although near the peritoneal reflection, the accuracy of tumour direction determination was rather high. However, as tumour size increased, it became more difficult to determine tumour direction. Nevertheless, the peritoneal reflection could be used to determine directions accurately for small size tumours less than 4 cm.

In a Dutch study, it was suggested that upper third rectal cancer be treated like colon cancer^18^, and in a Dutch trial, no significant difference was found between a radiotherapy plus surgery group and a surgery only group in terms of local recurrence rates in upper rectal cancer.^19^ Lopez-Kostner *et al.* suggested that treatment outcomes for rectal cancer located 10 to 15cm above the anal verge are similar to those of sigmoid colon cancer.^20^ At this time, no complete answer can be reached regarding whether upper third rectal cancer should be treated like colon cancer or rectal cancer.^15^ Nevertheless, recently, preoperative concurrent chemoradiotherapy has gained acceptance for the treatment of mid and lower rectal cancer.^5^

## Conclusions

In conclusions, we believe that subdivision of the rectum by rectal MRI based on the location of the peritoneal reflection is more objective and anatomical than previously described methods, and that the more accurate information obtained regarding anatomic relations between rectal tumours and the peritoneal reflection aids treatment planning.

## Figures and Tables

**TABLE 1.** Demographics and clinical status of patients

		**N=54**	**% (range)**
Gender	M : F	32:22	59.3 : 40.7
Age (year)		62.2 ± 10.8	(41–84)
Height (cm)		161.0 ± 9.6	(137 – 175)
Weight (kg)		63.4 ± 12.3	(41 – 107)
BMI (kg/m^2^)		23.1 ± 3.1	(17.4 – 42.3)
Proportion of high preop. CEA		18	33.3
Proportion of high preop. CA19-9		5	9.2
OP Name	LAR	49	90.7
	APR	5	9.3
Cell type (differentiation)	Well	2	3.7
	Moderately	48	88.9
	Poorly	2	3.7
	Mucinous	2	3.7
TNM stage	0	2	3.7
	I	11	20.4
	II	17	31.5
	III	22	40.7
	IV	2	3.7
Tumour size (cm)		4.8 ± 2.5	(0.9–10)
No. of retrieved LNs		24.3 ± 15.9	(3 – 87)
Distance from anal verge (cm)		8.8 ± 3.5	(1–12)

Mean ± standard deviation, BMI=body mass index, preop. = preoperative, OP=operation, LN=lymph node, LAR= low anterior resection, APR= abdomino-perineal resection.

**TABLE 2.** Location of rectal cancer with respect to peritoneal reflection by radiologic and operative findings

**No. of case**		**By surgeon**			
		Above PR	On PR	Below PR	Total
By radiologists	Above PR	29	1	0	30
	On PR	2	9	2	13
	Below PR	0	0	11	11
	Total	31	10	13	54

PR = peritoneal reflection

**TABLE 3.** Accuracies of predicting tumour directions and locations with respect to the peritoneal reflection

	**Prediction of peritoneal reflection**		**Prediction of tumour direction**	

	**Accuracy (%)**	**P**	**Accuracy (%)**	**P**
Gender		0.092		0.561
Male (n=32)	**90.9**		43.8	
Female (n=22)	**87.5**		45.5	
BMI		0.528		0.197
Low (<20 kg/m^2^) (n=7)	**88.0**		48.3	
Normal (20–25 kg/m^2^) (n=36)	**91.6**		45.1	
High ( > 25 kg/m^2^) (n=11)	**86.3**		43.0	
Relationship with PR		0.061		0.076
Above PR (n=31)	**93.5**		38.7	
On PR (n=10)	**90.0**		66.7	
Below PR (n=13)	**84.6**		46.2	
Tumour size (cm)		0.394		0.462
0∼1.9 (n=9)	**88.9**		66.7	
2.0∼3.9 (n=12)	**91.7**		58.3	
4.0∼5.9 (n=15)	**93.3**		46.7	
6.0∼7.9 (n=11)	**100.0**		36.4	
8.0∼10.0 (n=7)	**57.1**		28.6	

BMI = body mass index, PR = peritoneal reflection
